# Overview of digital health teaching courses in medical education in Germany in 2020

**DOI:** 10.3205/zma001476

**Published:** 2021-04-15

**Authors:** Jana Aulenkamp, Marie Mikuteit, Tobais Löffler, Jeremy Schmidt

**Affiliations:** 1 Universitätsklinikum Essen, Klinik für Anästhesiologie und Intensivmedizin, Essen, Germany; 2 Medizinische Hochschule Hannover, Hannover, Germany; 3 Eberhard-Karls Universität Tübingen, Tübingen, Germany; 4 Bundesvertretung der Medizinstudierenden in Deutschland, Berlin, Germany; 5 Ruprecht-Karls-Universität Heidelberg, Heidelberg, Germany

**Keywords:** education, digital health, digital competencies, digital medicine, digital teaching, eLearning, medical informatics

## Abstract

**Objective: **The digitalization of the healthcare system poses new challenges for physicians. Thus, the relevance of learning digital competencies (DiCo), such as dealing with data sets, apply telemedicine or using apps, is already growing in medical education. DiCo should be clearly separated from digitized teaching formats, which have been increasingly used since the COVID 19 pandemic. This article outlines the faculties in Germany where DiCo are already integrated into medical education.

**Methods: **Courses with DiCo as teaching content were collected by a literature research on Pubmed and Google as well as by contacting all dean's offices and other persons responsible for teaching at German medical faculties. The courses were summarized in a table.

**Results: **In a first survey, 16 universities were identified that offer courses on DiCo. In the elective area at the universities, 17 courses and in the compulsory area eight courses could be identified. The scope and content of the courses diverged between compulsory curricula, integrated courses of different lengths, and elective courses that are one-time or longitudinally integrated. The topics taught are heterogeneous and include fundamentals of medical informatics such as data management on the one hand and a collection of e.g. ethics, law, apps, artificial intelligence, telemedicine and robotics on the other hand.

**Conclusion: **Currently, only some German medical faculties offer courses on DiCo. These courses vary in scope and design. They are frequently part of the elective curriculum and only reach some of the students. The possibility of embedding DiCo in the already existing cross-sectional area appears limited. In view of the ongoing digitalization of healthcare, it is necessary to make future courses on DiCo accessible to all medical students. In order to drive this expansion forward, the implementation of the new learning objectives catalogue, in which DiCo are integrated, a network formation, a teaching qualification as well as the involvement of students is recommended.

## Zusammenfassung

**Zielsetzung: **Die Digitalisierung des Gesundheitswesens stellt Ärztinnen und Ärzte vor neue Herausforderungen. Somit wächst die Relevanz des Erlernens von Digitalen Kompetenzen (DiKo), wie z.B. Kenntnisse im Umgang mit Datenmengen, Telemedizin oder Apps, bereits im Medizinstudium. DiKo sind hierbei klar zu trennen von digitalisierten Lehrformaten, die seit der COVID 19-Pandemie vermehrt eingesetzt wurden. Dieser Artikel beschreibt, an welchen Fakultäten in Deutschland digitale Kompetenzen bereits in die Medizinische Ausbildung integriert sind.

**Methodik: **Über eine Literaturrecherche auf Pubmed und Google sowie durch den Kontakt zu allen Dekanaten und weiteren Lehrverantwortlichen deutscher medizinischer Fakultäten wurden Lehrveranstaltungen, die DiKo als Lehrinhalt aufweisen, gesammelt und in einer Tabelle zusammengefasst.

**Ergebnisse:** In einer ersten stichprobenartigen Übersicht wurden 16 Universitäten identifiziert, die Lehrveranstaltungen zu DiKo anbieten. Im Wahlpflichtbereich konnten 17 Universitäten und im Pflichtbereich acht Universitäten identifiziert werden. Der Umfang und die inhaltliche Ausgestaltung der Lehrveranstaltungen divergiert zwischen Pflichtcurricula, integrierten Lehrveranstaltungen verschiedener Größe und Wahlpflichtveranstaltungen, die einmalig oder longitudinal integriert sind. Die gelehrten Themen sind heterogen und umfassen einerseits Grundlagen der medizinischen Informatik, wie z.B. Datenmanagement und andererseits eine Sammlung aus z.B. Ethik, Recht, Apps, Künstlicher Intelligenz, Telemedizin und Robotik.

**Schlussfolgerung: **Aktuell werden nur an einem Teil der deutschen medizinischen Fakultäten Lehrveranstaltungen zu DiKo angeboten. Diese Veranstaltungen divergieren in Umfang und Ausgestaltung. Sie sind häufig im Wahlpflichtbereich angesiedelt und erreichen nur einen Teil der Studierenden. Eine Verankerung von DiKo im bereits vorhandenen Querschnittsbereich erscheint begrenzt. Angesichts der fortschreitenden Digitalisierung des Gesundheitswesens ist es notwendig, zukünftige Lehrveranstaltungen zu DiKo allen Medizinstudierenden zugänglich zu machen. Um diesen Ausbau zügig voranzutreiben wird die Umsetzung des neuen Lernzielkatalog, in den DiKo integriert sind, eine Netzwerkbildung, eine Lehrendenqualifikation sowie die Einbindung Studierender empfohlen.

## Introduction

The digitalisation of healthcare is a development that has been progressing for several years and is growing in importance. Easier access to medical knowledge, data-recording wearables, and telemedicine will permanently change healthcare in the next few years [1]. The first political basis has been set with laws for the provision of digital health applications, telemedicine and the electronic patient record [2], [3]. 

The majority of medical students has a positive view of the digitalisation of care, but at the same time students do not feel well prepared for this change in the healthcare system [4]. [5]. Also, health informatics and eHealth are not yet integrated throughout the field of advanced training [6]. Physicians have a special responsibility, for instance, in dealing with health apps, but they are not adequately prepared for this [7].

Dealing with these digital applications in everyday clinical life requires learning Digital Competencies (DiCo) [8], as training on the subject promotes acceptance [9] and such teaching should start early in education [10]. Even if students often use digital technologies in their everyday life, this does not imply that they can apply this in a professional healthcare setting [11]. The German Council of Science and Humanities recommended with regard to the “Masterplan Medizinstudium 2020” to integrate digitalisation as a central topic in medical education [12]. The German term “Digitale Kompetenzen” (Digital Competencies, short DiCo) is not used uniformly. The corresponding term “eHealth literacy”, established in the international context, describes an informed and self-confident handling of digital health information [13].

In medical education, a distinction must be made between the digitalisation of teaching methods (such as learning apps, PowerPoint, eLearning courses, etc.), and DiCo as teaching content. For DiCo, the German Society for Medical Informatics, Biometry and Epidemiology (GMDS) has published a catalog with learning objectives sorted in central topic areas as a guide [14]. In the National Competence-Based Learning Objectives Catalog for Medicine (NKLM), there are currently hardly any learning objectives that can be assigned to DiCo [8]. 

At present, there is no overview of the courses that are already offered on DiCo available. Therefore, it is uncertain to what extent future physicians are prepared for the developments in the digitalisation of healthcare. This article aims to present a descriptive inventory of the teaching of DiCo at medical faculties in Germany.

## Methodology

The research of DiCo projects was performed in three ways: via an online search, two queries of the deaneries of all medical faculties in Germany, and a survey of the “digital competencies” working group of the NKLM/GK process.

First, a simple search via PubMed and Google using the keywords “digital health”, “digital competencies”, “digital literacy”, “medical school”, “medical education” and the corresponding German words was conducted, for papers on projects at German universities, colleges, and medical faculties.

Simultaneously, the deaneries of all German medical faculties as well as all members of the NKLM/GK working group “Digital Competencies” were contacted by mail and asked to name corresponding concepts, projects and courses. The deaneries were contacted under the general e-mail address and the persons most likely to be responsible in each case were approached, for example curriculum officers, digitalisation officers and officers for the timetable, if their addresses were found on the website. Telephone interviews were conducted in some cases. The deans of studies were queried in November 2019 and again as part of the review process in June/July 2020. The members of the NKLM/GK working group were contacted, as they were assumed to have expertise in the courses offered.

The overview in this article only includes courses which, in the view of the authors, deal with digitalisation in medicine and the teaching of digital competencies as teaching content. This selection was based on the GMDS learning objectives catalogue on medical informatics competencies for physicians [14]. Courses that use digitalisation as a teaching method but teach competencies that are primarily independent of digitalisation in healthcare were excluded from this overview. Similarly, courses that were in the planning phase but not implemented at time of assessment were excluded. The teaching of DiCo which are not officially part of a course and comprise less than one teaching unit (UE, equivalent to 45 min) were also excluded.

The courses have been listed in a table and broken down by scope, content, elective or compulsory, and examination type.

## Results

**Figure 1:**
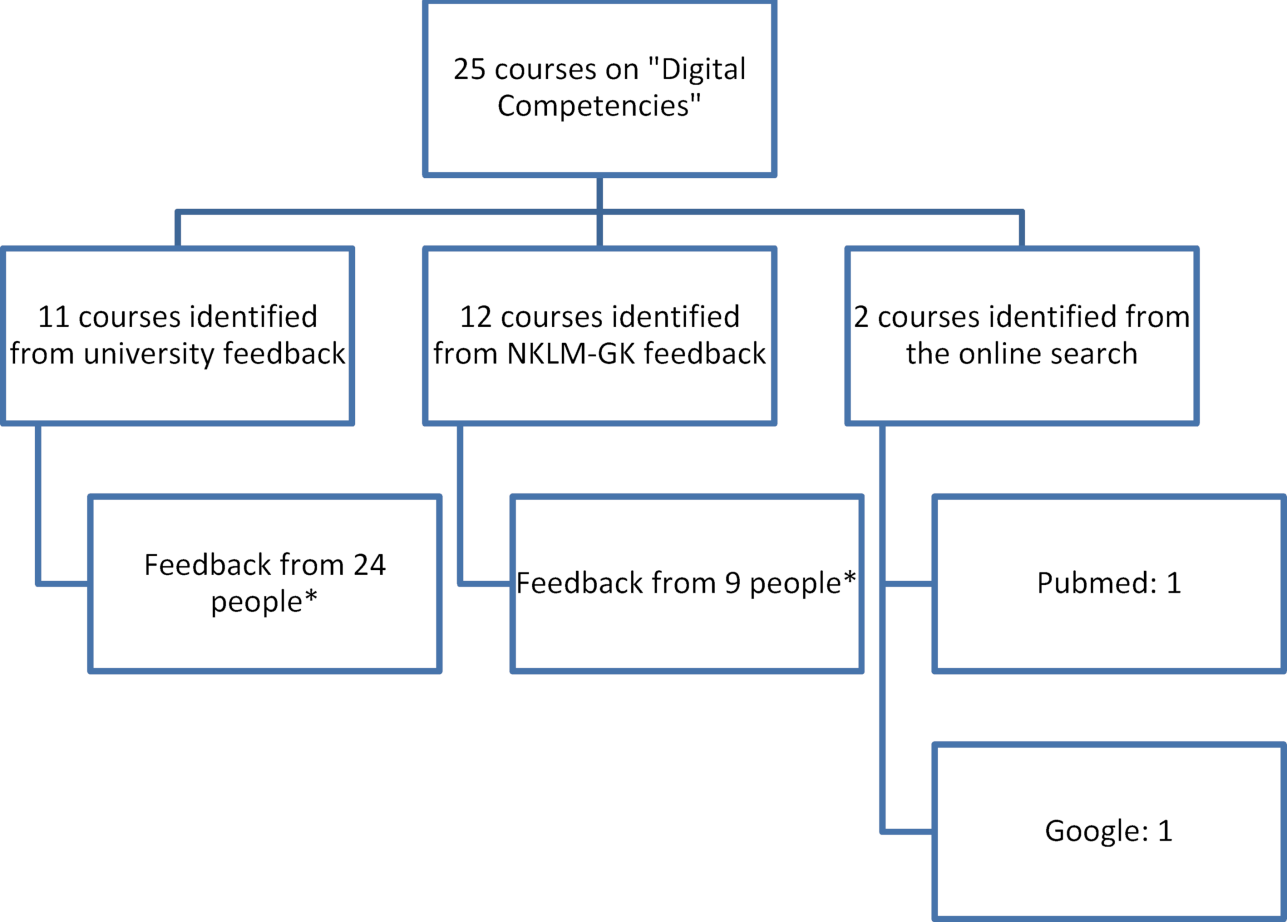
Distribution of research results for the overview "Digital Competences" in medical schools 2020 (*= One person was able to report back on several courses, NKLM-GK group = "Digital Competences" working group of the further development process for the National Catalogue of Learning Goals for Medicine and the subject catalogues of medical studies)

24 faculties and 9 members of the "Digital Competencies" working group of the NKLM/GK-process reported back. One course was found via Pubmed and one via Google search (see figure 1 (Fig. 1)). In total, 25 projects could be included in the overview. The main reason for excluding courses was that they featured digitisation of teaching materials and teaching methods but did not address the topic of “digitalisation” as teaching content. 

According to our survey, there are currently 16 universities that have integrated DiCo into the curricula of medical education (see attachment 1 ). We were able to list 17 courses in the elective area and eight courses in the compulsory area. Of all the courses, only three extend longitudinally over several semesters. The extent of integration at the universities diverges. For example, the learning objectives for DiCo are partly integrated into other elective courses for a few students (cf. Bochum) [15], to a lesser extent part of the compulsory curriculum via the cross-sectional area “Epidemiology, Medical Biometry and Medical Informatics” (EBI) (cf. Münster) [16], or established in a focused manner in extensive elective modules over several semesters (cf. UKE Hamburg) [17]. Most elective subjects are designed for 10-25 students, so that currently often only a small fraction of medical students is reached. Some subjects are only scheduled for a few hours, others contain 60 lessons (cf. Berlin) [9] or are combined with a clinical elective (cf. Giessen) [18].

In most cases, the courses cover a wide range of digital medicine. For example, they primarily include the following learning objectives:

- address the topic: reflecting on physician roles in the digital age,
- evaluate possibilities of using mobile apps as well as indications for using telemedicine,
- know machine learning and explain basic principles of neural networks,
- differentiate between legal and ethical aspects of the digitalisation of healthcare.

However, the content of the individual electives varies, so that further clustering and subdivision based on content or learning objectives was not possible. For example, in Halle, students are given the opportunity to practice interprofessional ward rounds in telepresence modules, using a tablet with video transmission [19]. In Mainz, students are using various apps, such as anamnesis support, and reflecting on this [20]. 

In the compulsory area, medical informatics with the fundamentals of data management, workplace systems and telemedicine is established at some universities, primarily via the cross-sectional area EBI. The focus on the field of medical informatics could only be evaluated at a few locations. These topics are largely aligned with the GMDS learning objectives catalogue. For example, at the Hannover and Münster campuses, practical trainings prepare students for clinically relevant activities, such as workplace systems, online research, or the potential impact of digitalisation on health care [16], [21]. The extent to which the courses are intended to enable medical students to develop their own ideas and to deal not only with current but also future circumstances was evident in individual cases (cf. Berlin and Marburg) [9].

## Discussion

This first overview of courses on digital competencies at German medical faculties shows that several faculties have already integrated the subject, but mainly in the elective area so far.

The courses vary widely in scope and design, so that a classification based on definable content criteria was not possible. The current elective courses already cover a wide range of subjects and mainly provide a general overview of current digital trends in healthcare, such as digital health applications, robotics or new healthcare models. In some cases, students are given the opportunity to reflect on ethical, legal or political issues. Due to the very heterogeneous teaching content and the different presentation of the content, no content clustering was done. 

Unfortunately, the extent to which DiCo are established within the framework of the already existing cross-sectional area of EBI could only be partially determined. There was little feedback from the deaneries in this case. In Münster, for example, the basics of medical informatics are not only mentioned in a lecture, but also deepened in an application-related way in a seminar. Otherwise, it can be assumed, that DiCo are currently not sufficiently integrated into the cross-sectional areas. On the one hand it is due to the poor level of information of medical students on digitalisation [4]. [5] and on the other hand it is due to the increasing demands for more competencies in digital medicine [8], [14], [21]. The GMDS has made efforts to adapt the content of the cross-sectional area since 2012 [22], but these developments do not appear to be sufficient, so that the GMDS and the Society for Medical Education are calling for a national initiative [8]. There is potential for the further expansion of the already existing cross-sectional area. In the future, longitudinal implementation could also improve learning outcomes and linkage with clinical subjects is suggested [9], [23].

The low status of courses on DiCo contrasts with the change in the reality of care due to digitalisation, for which students should be prepared through basic knowledge up to the development of their own attitude. At the same time, training is an opportunity to implement new technologies in the healthcare system in a meaningful way [22].

## Challenges and limitations

The term “digitalisation” is a mixture in its meaning for university teaching. elearning platforms, PC exams or simulation programs, i.e. digitized teaching, are equated with the teaching of digital competencies. The reason for this may lie both in a translation of “digital health” or “eHealth literacy” that is not yet used in everyday language, and in definitions of the associated content for medical education that are also not yet standardised internationally. “Digital competencies” is hardly used in English-language literature. The more common term “digital literacy” is used partly as an overarching collective term and partly as one of many components of digital health for the subarea of accessing and using knowledge and sources, often overlapping with “digital health literacy” and “e-health literacy” [24], [25]. Online searches likewise proved difficult because of the inconsistent scientific terminology addressed. In the United States, despite many existing chairs and initial certificates, integration into medical education is still mostly limited to electives and based on inconsistent content [26].

In Germany, the term “digital competencies” has become established [8], [21], [26], [27], [28]. We consider this to be suitable for providing a distinction from digitized teaching. 

This inconsistent term complicates the question of a uniform definition of learning objective content: What do digital competencies include and are they covered as completely as possible in the curriculum? The GMDS has presented a catalogue of learning objectives for the basic structure of the DiCo [14], and there are also competence frameworks in the international context, for example the “eHealth capabilities framework” of an Australian research group [23] or the “Competence Framework” of the EU [29]. In the working group “digital competencies” as part of the further development of the NKLM and the GK, the subject area is being put together in learning objectives and is to be published in the near future. Due to the obligation of the NKLM for faculty teaching and the GK for examinations, which is specified in the Master Plan for Medical Studies, many faculties will develop or deepen their teaching on the basis of the competencies and learning objectives formulated there. 

Since the response rate of the deaneries and working group members was approximately 65%, it is possible that not all existing projects are listed. In addition, it should be mentioned that not all teaching projects are published and the homepages of the faculties are very differently designed, so that the teaching contents of the modules were sometimes difficult to identify. Furthermore, some courses were excluded from our analysis (duration<1 lesson of 45min). In addition, it appeared that the deaneries found it difficult to distinguish between digitisation of teaching and DiCo as teaching content in their feedback, despite emphasising it several times. This should be viewed critically in terms of awareness of the problem. 

Therefore, this analysis can only be understood as a first overview and encourage future exchange and networking on the DiCo courses. For a better qualitative overview of the contents, a survey through direct interviews with the teachers about the concrete schedule of the courses is advisable. 

## Recommendations to the faculties

Many universities have already reported that they are currently developing new modules on digital competencies or expanding existing ones. In the development of a curriculum on DiCo, the following recommendations emerge from the authors’ point of view:

An inventory at the individual faculty to bundle the various courses, as for example in Hanover, is recommended [21]. Courses and their modules should be linked in the curriculum to demonstrate the diverse facets of the subject completely, but also not duplicate aspects within the overall programme. The use of the new NKLM as a basis for DiCo courses is indispensable. Integration into already existing teaching formats or clinical disciplines is possible, flexible formats that are adaptable should be chosen [23]. Furthermore, technical resources must be made available [30]. 

In order to gain further scientific expertise in this area, there is the option of founding own chairs, or starting new master’s programs on medical informatics, medical management or even digital medicine (Technische Hochschule Mittelhessen). Networking beyond one's own faculty for competence transfer and mutual benefit through existing pilot projects can be the key to faster and cross-faculty embedding [9], [31]. Currently, eleven universities, including several medical faculties, are working on a large collaboration (HighMed Teaching Program [32]) to create a learning platform on DiCo. Individual modules are to be offered with face-to-face sessions across professions. Such mono- or interprofessional collaborations between institutions and specialties offer great opportunities for rapid and collaborative progress [8] and are important for different professional groups across the health care team to understand each other’s systems requirements [30]. It is also feasible to involve e-health start-ups, health insurance companies, or interdisciplinary teaching with computer scientists, health services researchers, or pharmacists.

In this context, the training of teachers must also be considered in addition to the training of students. In addition to expanding the chairs, such as the new medical faculty in Bielefeld, which is developing its own chair for didactics, digitalization and interprofessionalism, further training programs must be initiated. In our view, the integration of committed students, who often have independent content expertise, into teaching is also advisable [33]. For example, in Dresden, the curriculum certified by the Saxony Medical Association, in which students acquire DiCo together with physicians and participants from informatics, was initiated by students. 

Overall, the teaching of digital competencies at the medical faculties is still being developed. At some campuses, digital competencies are already taught to some of the students, mostly as elective courses. Due to the increasing digitalization of healthcare, an expansion of courses and an increased inclusion in the compulsory curriculum on digital competencies appears future-oriented. Therefore, it is recommended for medical faculties to take a look at existing teaching elements, to implement DiCo learning objectives based on the new NKLM, to professionalize (chairs, continuing education) to network and to integrate students in the process. Students should already be prepared for the digital transformation of healthcare reality as part of their education.

## Competing interests

The authors declare that they have no competing interests. 

## Supplementary Material

Overview of teaching projects at medical faculties in Germany
