# Supplementary material for: Overview of digital health teaching courses in medical education in Germany in 2020
Source: GMS J Med Educ. 2021 Apr 15;38(4):Doc80. doi: 10.3205/zma001476 (PMC8136344; doi:10.3205/zma001476)
Supplement: Overview of teaching projects at medical faculties in Germany [file JME-38-4-80-s-001.pdf]

Attachment 1: Overview of teaching projects at medical faculties in Germany

	University	Course name	Scope and special features	Exam	Course content
elective courses	Charité Berlin	Digital Health: From the App to the smart hospital	60 lessons within 3 weeks	Quiz (not graded). Participation in co-productions & group work as part of the mini-hackathon	Chances and limits of the use of digital procedures for the doctor-patient relationship and practice<br>- Be able to reflect on possible uses of medical apps<br>- Be able to explain the legal, ethical and socio-political framework of digital medicine<br>- Telemedicine & apps
	Charité Berlin	Artificial intelligence in medicine	60 lessons within 3 weeks	Develop artificial intelligence; review of application idea, short presentation followed by subsequent discussion and structured feedback	Students should...<br>- understand basic principles of Artificial Intelligence and be able to write programs in Python independently<br>- learn about medical applications of artificial intelligence and be able to independently develop new questions and possible applications<br>- get to know the practical, legal and ethical framework and be able to name and reflect on the potentials and challenges of artificial intelligence
	Ruhr University Bochum	Medicine 4.0	part of elective course, approx. 11 lessons	term paper	- Lean Management<br>- Error Management<br>- Agile Management<br>- Digitalisation and its impact on the work environment

Attachment 1 to: Aulenkamp J, Mikuteit M, Löffler T, Schmidt J. *Overview of digital health teaching courses in medical education in Germany in 2020*. GMS J Med Educ. 2021;38(4):Doc80. DOI: 10.3205/zma001476

Faculty of Medicine Carl Gustav Carus	Clinicum Digitale	Impulse day and in-depth curriculum of 40 hours, interdisciplinary event for medicine and informatics	not determinable	intensive days<br>- basic knowledge of computer science or resp. medicine<br>- legal, economic and also ethical aspects<br>module days:<br>- Human Interface & Robotics<br>- Data Science & Machine Learning<br>Perspective Days<br>- interdisciplinary work or applied digitalisation
Albert Ludwig University of Freiburg	Medical Informatics	2 Semester hours weekly	written presentation	- Information Technology<br>- data protection<br>- Documentation<br>- Artificial intelligence
Justus Liebig University Giessen	Digital medicine, eHealth and telemedicine	Various compulsory modules and elective modules over several semesters + 30 days of clinical traineeship with a focus on digital medicine	not determinable	- Information Systems<br>- Communication Systems<br>- Artificial intelligence, image processing and pattern recognition<br>- Robotics<br>- Law and ethics
Martin Luther University Halle-Wittenberg	Teleconsil - interprofessional communication in the digital age	approx. 4 lessons, interprofessionally affiliated with the Skillslab	not determinable	Medical students and nursing students go through and reflect on an interprofessional televisit in nursing homes
Medical Center Hamburg-Eppendorf	Digital Health	Track in the model study program, two weeks of instruction in the preclinical phase,and five two-week in-depth seminars in the 5th-9th semesters with a final course paper in the 10th semester	Multiple choice exam or short presentation	Topics of the modules:<br>1.) Telemedicine<br>2.) Robotics, automation and VR & AR<br>3.) Artificial Intelligence<br>4.) Big Data: omics and biomarkers for personalized medicine<br>5.) Smart Medical Devices and Health Apps

	Medical School Hamburg	Medicine in a digital world	28 lessons elective course	Review of an app or AI application (text and presentation)	- telemedicine<br>- digital health applications<br>- "Clinical Decision Support" based on Artificial Intelligence and Data Literacy
	Hannover Medical School	Digitization of Medicine - Acquisition of Basic Competencies in Data Literacy	approx. 11 face to face lessons and approx. 27 online lessons	Oral examination	Data Literacy:<br>- Conceptual Framework<br>- Data Collection<br>- Data Management<br>- Data Evaluation<br>- Data Application
	Ruprecht Karl University of Heidelberg	Digital medicine	four dates with a total of approx. 31 lessons	not determinable	- What will medicine of the future look like?<br>- Apps and chatbots<br>- Data privacy<br>- Doctor-patient relationship<br>- Machine learning, blockchain
	Johannes Gutenberg Universsity Mainz	Medicine in the digital age	Elective course for one week with approx. four face to face lessons each as well as e-learning unit since summer semester 2017	existing	1.) Digital doctor-patient communication and social networks<br>2.) "Smart devices" and medical apps<br>3.) Tele emergency physician, teleradiology, telemedicine<br>4.) "Virtual reality," "augmented reality" and computer-assisted surgery<br>5.) Individualized medicine and BigData
	Philipps University of Marburg	Image analysis and AI methods for medical professionals	not determinable	Project and oral exam	- Development of concepts and IT potentials<br>- Human vs. machine "seeing",<br>- AI, deep learning, artificial creativity, AI in research,<br>- discussion of scenarios
	Ulm University	Fit for digitization in the healthcare system	elective preclinical	Term paper	- Explain and evaluate principles relating to medical confidentiality, data protection in the health care sector and copyright<br>- transfer principles to new problem cases in professional practice and assess the associated opportunities and risks<br>- practice responsible handling of data and media

	Witten/Herdecke University	Digital Literacy	five ECTS	Project	EU Digital Literacy Framework with the following content:<br>1) Information and Data Literacy<br>2) Communication and Collaboration<br>3) Digital Content Creation<br>4) Privacy<br>5) Problem Solving
	Witten/ Herdecke University	Digital Medicine	two-four ECTS	Presentation/essay	Current topics in the digital transformation of healthcare
	Witten/ Herdecke University	Digital transformation - master plan	two ECTS	Online Multiple Choice Exam	Digital transformation of society, the economy and the healthcare system
compulsory courses	Martin Luther University Halle-Wittenberg	Digitisation Curriculum	six lessons, 5th semester Human Medicine	none / only reflection	Introduction to the topic of digitalisation in medicine<br>Hands-on and reflection on the topics:<br>- Medical apps, robotics, VR/AR applications, 3D printing<br>- Reflection of possible applications in medicine, limits, risks and opportunities<br>- Counseling skills for patients on the use of medical apps
	Hannover Medical School	Medical Informatics (Lecture and Seminar)	20 lessons lecture, six lessons seminar	Multiple Choice Exam	- Basics of medical informatics such as image processing and med. information systems<br>- Digitization in prevention, diagnostics and therapy, in research, science and teaching as well as in the organization of the health care system<br>- Data preparation and analysis (Excel), Big Data & Data Mining

	Hannover Medical School	Seminar Teleneurologie	three lessons integrated as part of neurology	integrated questions in module exam	- Communication technology & data transfer<br>- requirements for telemedicine systems<br>- ethical and legal responsibility<br>- Diagnosis, use of additional tools (e.g. apps, smart devices...)
	Ruprecht Karl University of Heidelberg	Seminar Teleneurology	Part of the cross-sectional area: "Epidemiology, medical biometry and medical informatics".<br>approx. seven lessons and approx. three lessons practical training	Part of a written exam	Importance of systematic data collection and data management for both the quality of medical care and the economic design of care processes.<br>Lecture:<br>- Theory of data, images and biosignals.<br>Practical Training:<br>- Coding, classification and research
	University of Münster	Medical Informatics	1st semester: Introductory course<br>5th semester: Lecture and practical training	Part of a written exam	Lecture, I.A:<br>- Information management<br>- telematics<br>- Biomedical Informatics<br>Practical Training:<br>- clinical workplace system such as PACS, ICD,/OPS<br>- electronic charting, coding<br>- Pubmed research strategies
	University of Lübeck	Medical Informatics	Part of the cross-sectional area: "Epidemiology, medical biometry and medical informatics".<br>Lecture (two semester hours per week, 30 lessons) and practical exercises in small groups (two semester hours per week, 30 lessons)	Multiple Choice Exam	- Overview of the field of medical informatics and basic principles of informatics<br>- Medical documentation and clinical information systems<br>- Hospital management<br>- Coding of diagnoses and procedures (ICD-10, OPS)<br>- DRG case classification system<br>- Medical image processing<br>- Telemedicine, health telematics and data protection

	Philipps University of Marburg	cross-sectional area	Medical informatics part of the compulsory course cross-sectional area one	Multiple Choice Exam	- Basic knowledge from the field of med. informatics<br>- Data protection<br>- Digital applications in relation to diagnosis and surgery coding (Diacos) to increase drug therapy safety and evidence-based medicine (UpToDate)
	Witten/ Herdecke University	Quo vadis medical studies? Digital care as a central component of training	Integrated:<br>Personality development in the 1st-10th semesters via the study fundamental basics<br>Medical informatics in the 2nd-4th semesters (methodology).<br>Scientific work in the 6th &10th semesters (open topics)		Digital Medicine - How will data change the way we treat?<br>- Digital Literacy<br>- Economic, ethical and political implications of the digitalisation of health care<br>- Minimizing risks, exploiting opportunities

Attachment 1 to: Aulenkamp J, Mikuteit M, Löffler T, Schmidt J. *Overview of digital health teaching courses in medical education in Germany in 2020*. GMS J Med Educ. 2021;38(4):Doc80. DOI: 10.3205/zma001476
